# Supplementary material for: CCDC88B is required for pathogenesis of inflammatory bowel disease
Source: Nat Commun. 2017 Oct 13;8:932. doi: 10.1038/s41467-017-01381-y (PMC5640600; doi:10.1038/s41467-017-01381-y)
Supplement: Supplementary file 1 — Supplementary information [file 41467_2017_1381_MOESM1_ESM.pdf]

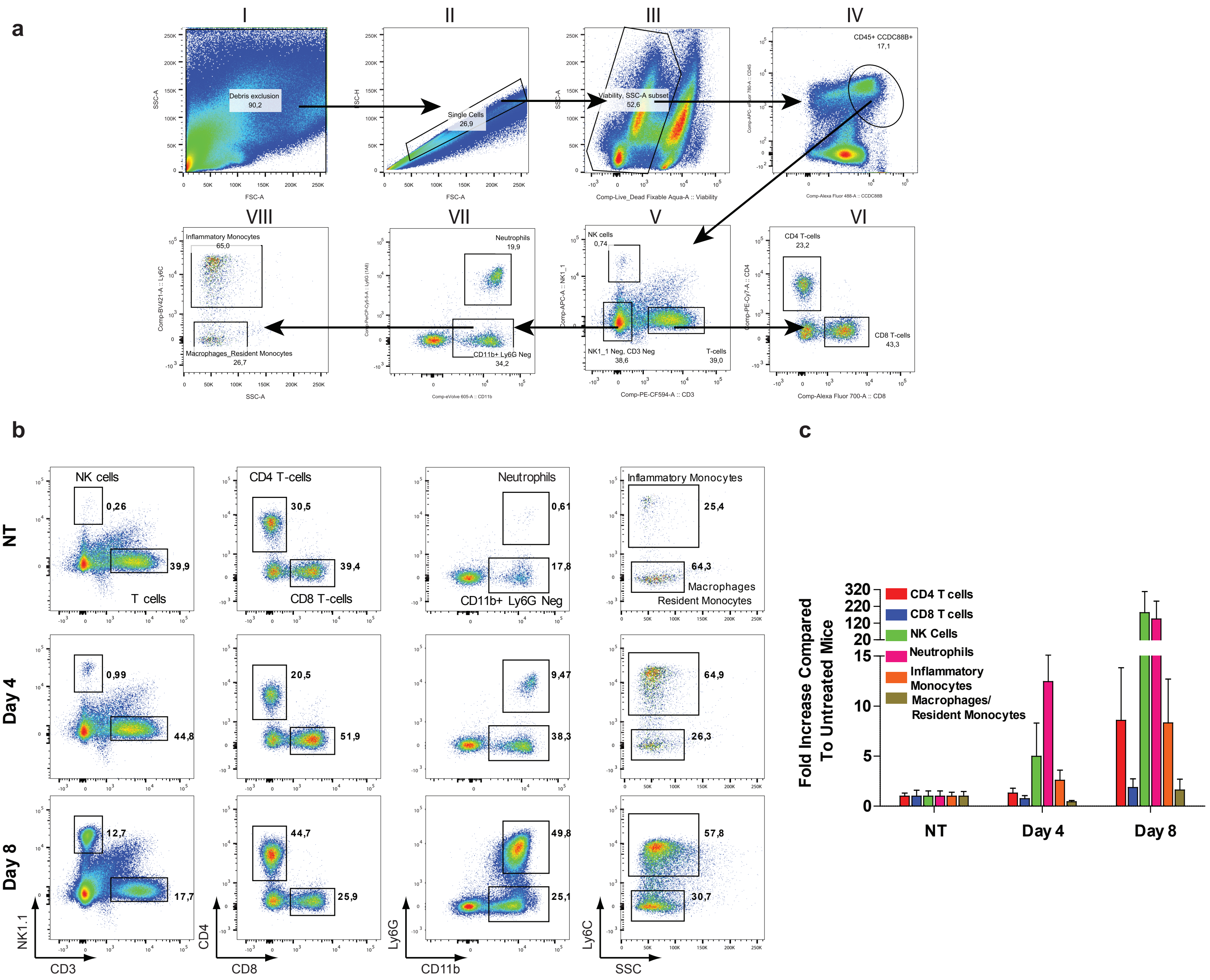

Supplementary Figure 1: Ccdc88b expression in lamina propria lymphocytes following DSS treatment.

(a) Flow cytometry gating strategy, single-cell suspensions from the lamina propria were stained intracellularly for Ccdc88b along with the indicated extracellular markers. Cells were gated to exclude cell debris (I) then single cells were gated to exclude aggregates (II), followed by gating the viable cells (III) CD45+CCDC88B+ cells are gated on (IV), NK and T cells are then gated on (V) and CD4 and CD8 T cells are gated on (VI), neutrophils on (VII) and monocytes on (VIII). The same gating strategy was used in all time points. **(b)** Representative FACS analysis for CD45+CCDC88B+ staining further examined for the indicated cells subsets in Not Treated (NT) (n=3) and DSS treated mice (n=4 for both day 4 and 8). **(c)** Fold increase in indicated cell subsets numbers from colons of DSS treated mice compared to NT mice.

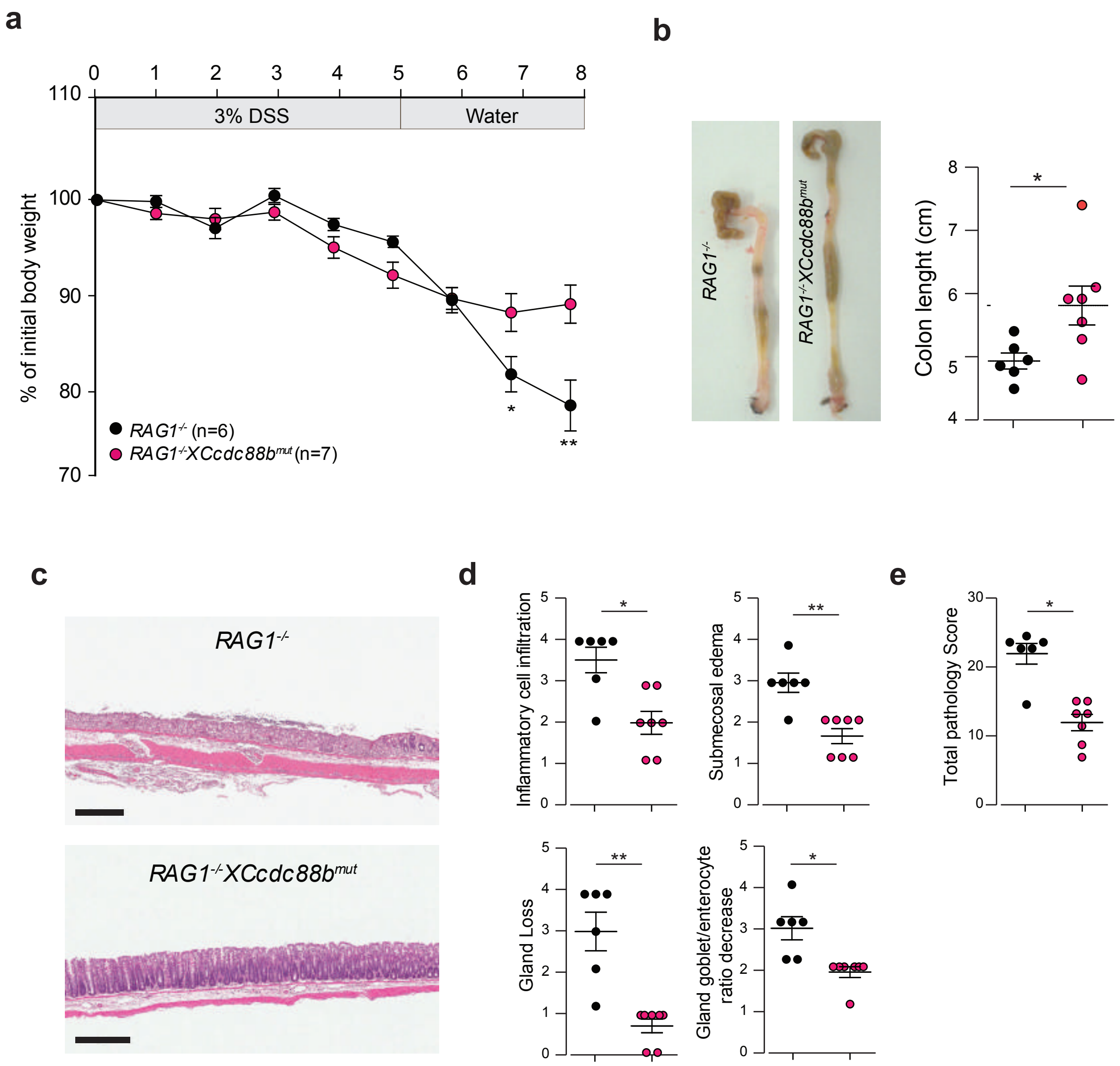

Supplementary Figure 2: Ccdc88b is required for DSS induced colitis pathology in the absence of adaptive immune cells.

Rag1$^{-/-}$ (n= 6) or *Rag1*$^{-/-}$*x Ccdc88b*^{mut} (n=7) mice were given 3% (w/v) DSS in the drinking water for 5 days, followed by water until day 8. **(a)** Body weight loss is expressed as percent of initial weight ± SEM. *p<0.05 and **p<0.01 (Mann-Whitney test). **(b)** Representative images of colons from *Rag1*$^{-/-}$ or *Rag1*$^{-/-}$*x Ccdc88b*^{mut} mice at day 8, and quantification of effect of treatment on colon length ± SEM. *p<0.05 (two-tailed Student's t-test). **(c)** Hematoxylin and eosin (original magnification 10x) staining of colon sections from DSS-treated mice at day 8. Scale bars, 400 µm. **(d)** Histology scores from *Rag1*$^{-/-}$ or *Rag1*$^{-/-}$*x Ccdc88b*^{mut} mice at day 8 evaluating inflammatory cells infiltration, submucosal edema, gland loss and gland goblet/enterocytes ratio decrease ± SEM. *p<0.05 and **p<0.01 (Mann-Whitney test). **(e)** Total pathology score (0-24) at day 8 ± SEM. *p<0.05 (Mann-Whitney test). Results are representative of one experiment.

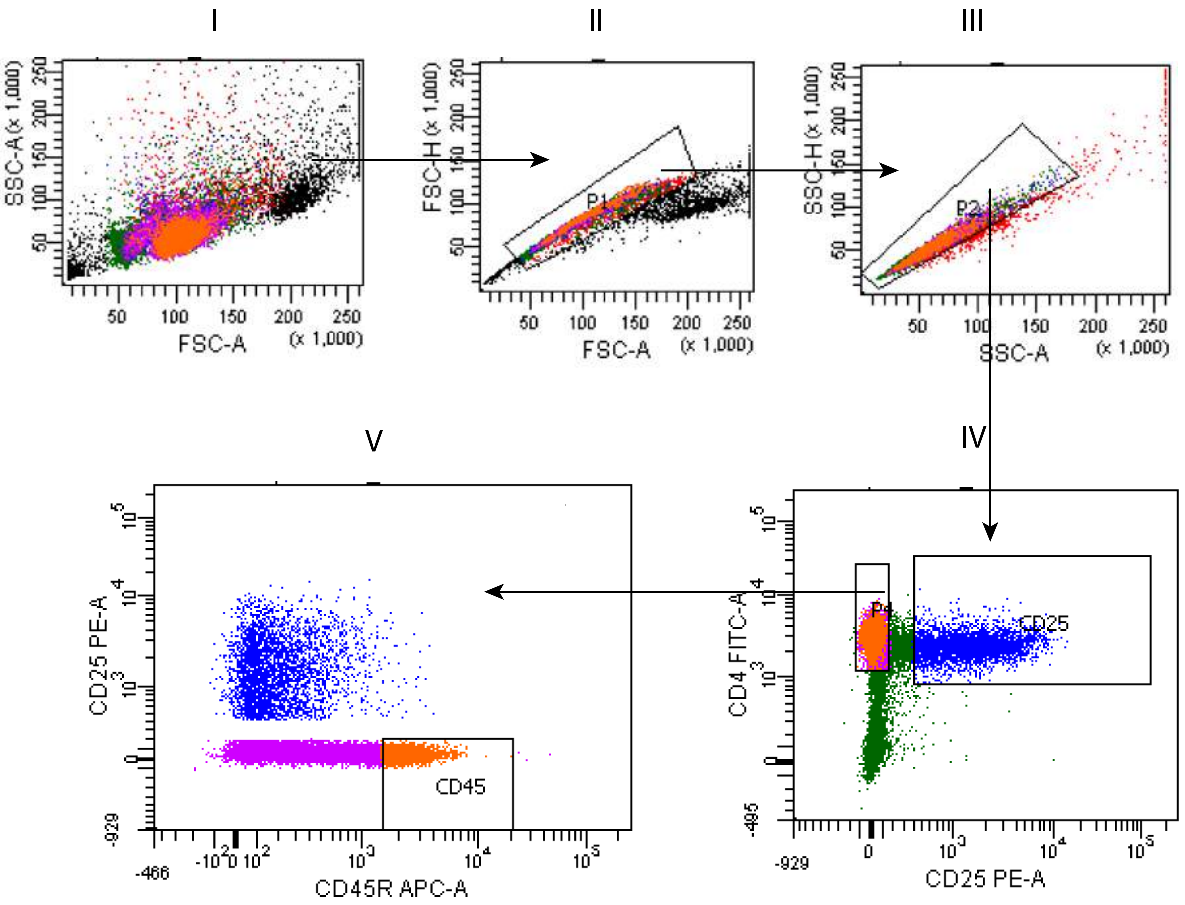

Supplementary Figure 3: Gaiting strategy for the sort of naive CD4⁺ CD45RB^hi T cells. Single suspension of enriched CD4⁺T spleen cells were stained with anti-CD4, anti-CD25 and anti-CD45RB antibodies. Total cell populations were gated (I) then single cells were gated to exclude aggregates (II and III), then CD4⁺ CD25⁻ T cells were gaited (IV) and CD4⁺CD45RB^hi were gated and sorted (V).

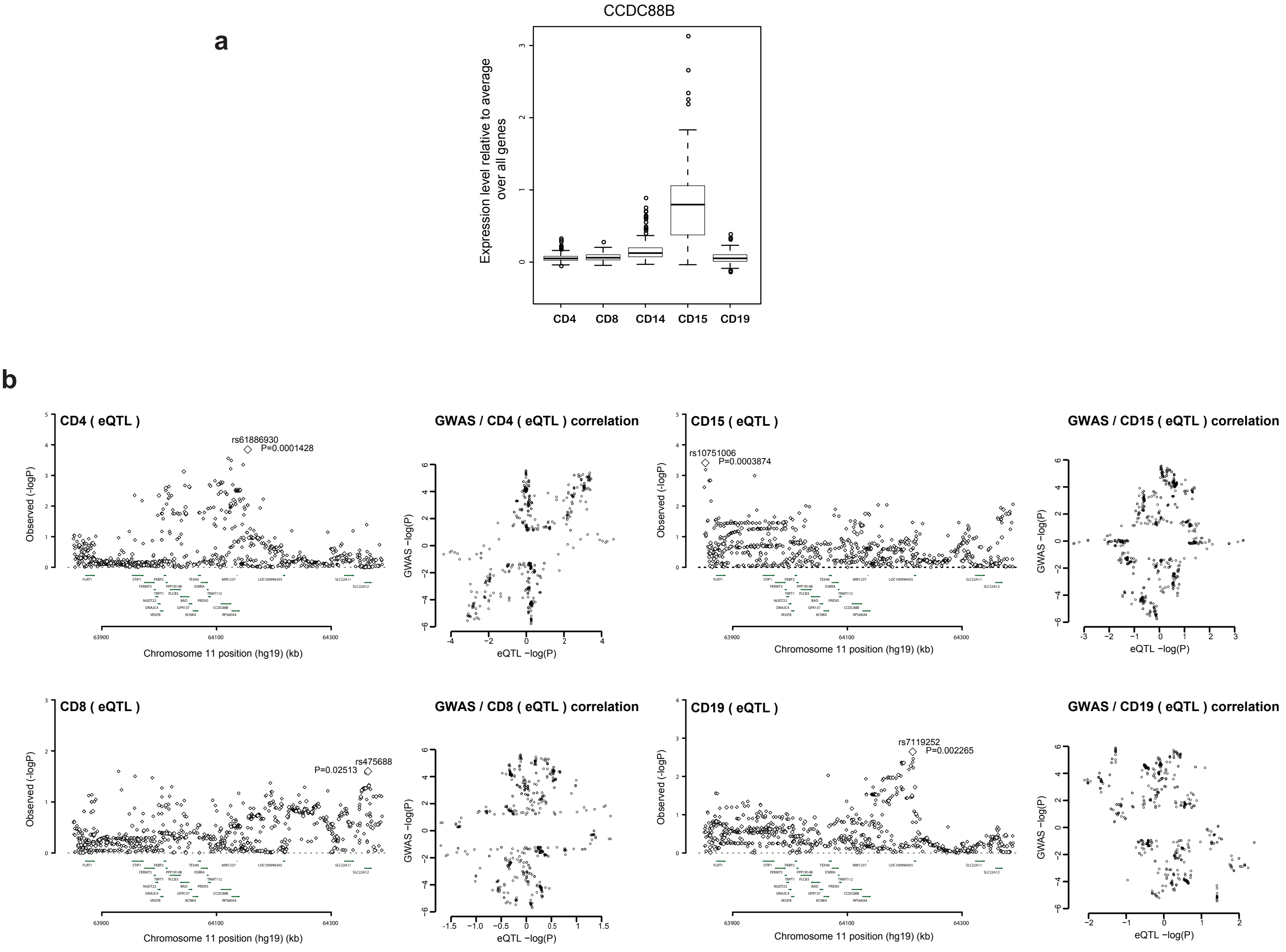

Supplementary Figure 4: CCDC88B mRNA expression in hematopeitic cell subsets and eQTL association studies.

(a) *CCDC88B* mRNA expression in CD4$^+$, CD8$^+$, CD14$^+$, CD15$^+$, and CD19$^+$ isolated from PBMCs of 300 healthy individuals as in **Figure 5**. **(b)** left, eQTL association pattern for CCDC88B expression in indicated cells subsets for the 600Kb window on 11q13 as in **Figure 5**. The maximal association signal SNP for each subset, using a logistic regression analysis [see Methods], is indicated. Gene names and positions are shown under each graph. Right, correlation between the disease risk and eQTL association patterns.

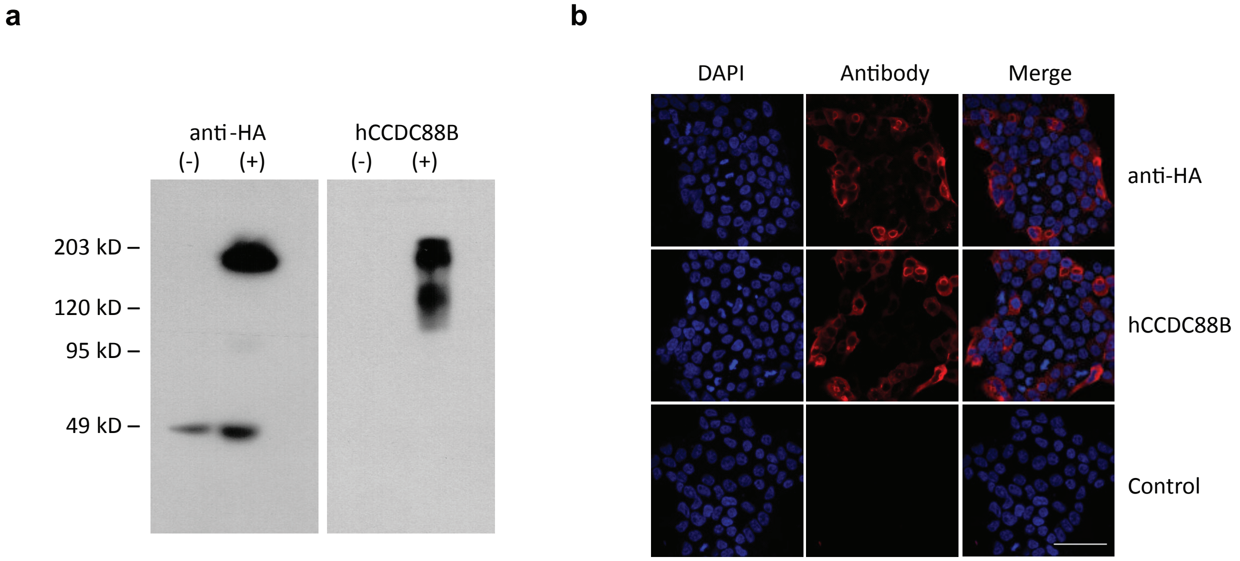

Supplementary Figure 5:Production and characterization of rabbit anti-human CCDC88B polyclonal antibody.

(a) Immunoblotting analysis of total cell extracts from HEK293T control cells (-) and HEK293T cells (ATCC, CRL-11268) stably expressing a full-length human *CCDC88B* (+) modified by the addition of an in-frame HA epitope tag (HA; indicated), and analysed with anti-HA (anti-HA) or anti-CCDC88B hyper-immune serum (anti-CCDC88B). Molecular mass markers are identified to the left of the blots. **(b)** Un-transfected control cells (bottom row), as well as HEK293T cells transfected with full length HA-tagged *CCDC88B* cDNA (top and middle rows) were stained with either anti-HA (top row) or anti-CCDC88B antiserum (middle row) and examined by immunofluorescence. The last column is a merge of images from the first and second columns. Bars, 50 μm for all images.

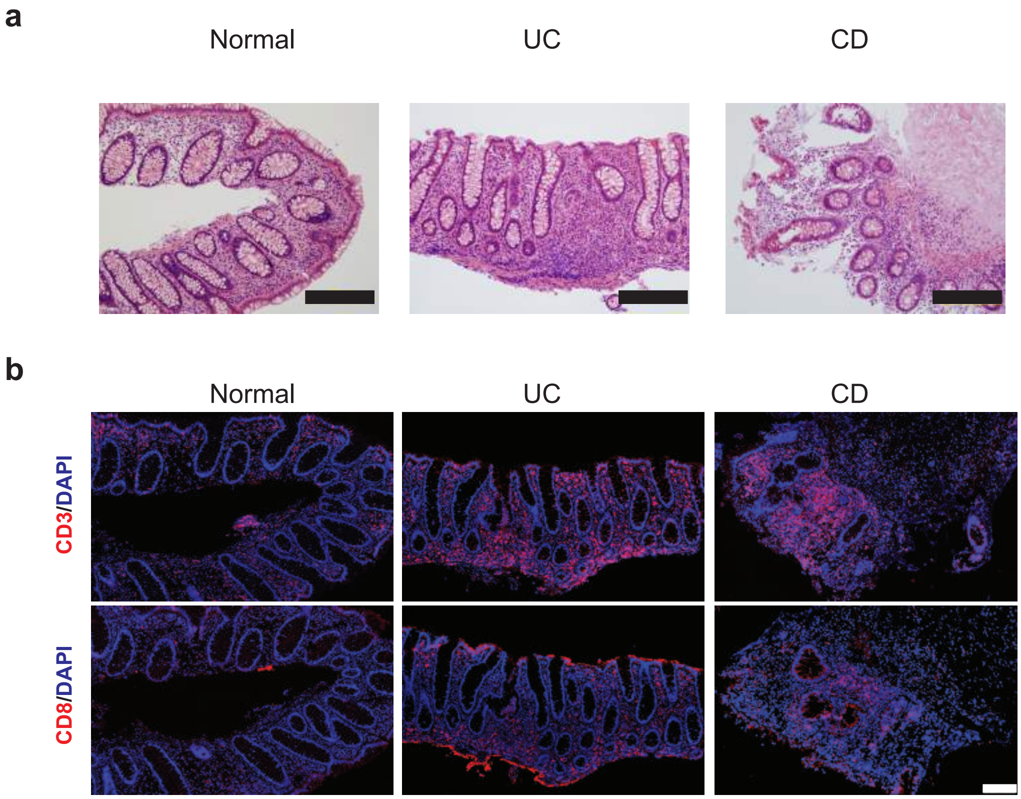

Supplementary Figure 6: Increased infiltration cells of CD3 and CD8 positive in the lamina propria of colon sections of UC and CD patients.
(a) Representative images of haematoxylin and eosin (original magnification 10x) staining of NL (n=2), UC (n=5) and CD (n=4) colon sections; scale bars, 100 µm. **(b)** Representative images of NL (n=1), UC (n=1) and CD (n=1) colon sections stained with anti-CD3 or anti-CD8 antibodies and counterstained with DAPI (original magnification 5x); scale bars, 100 µm.

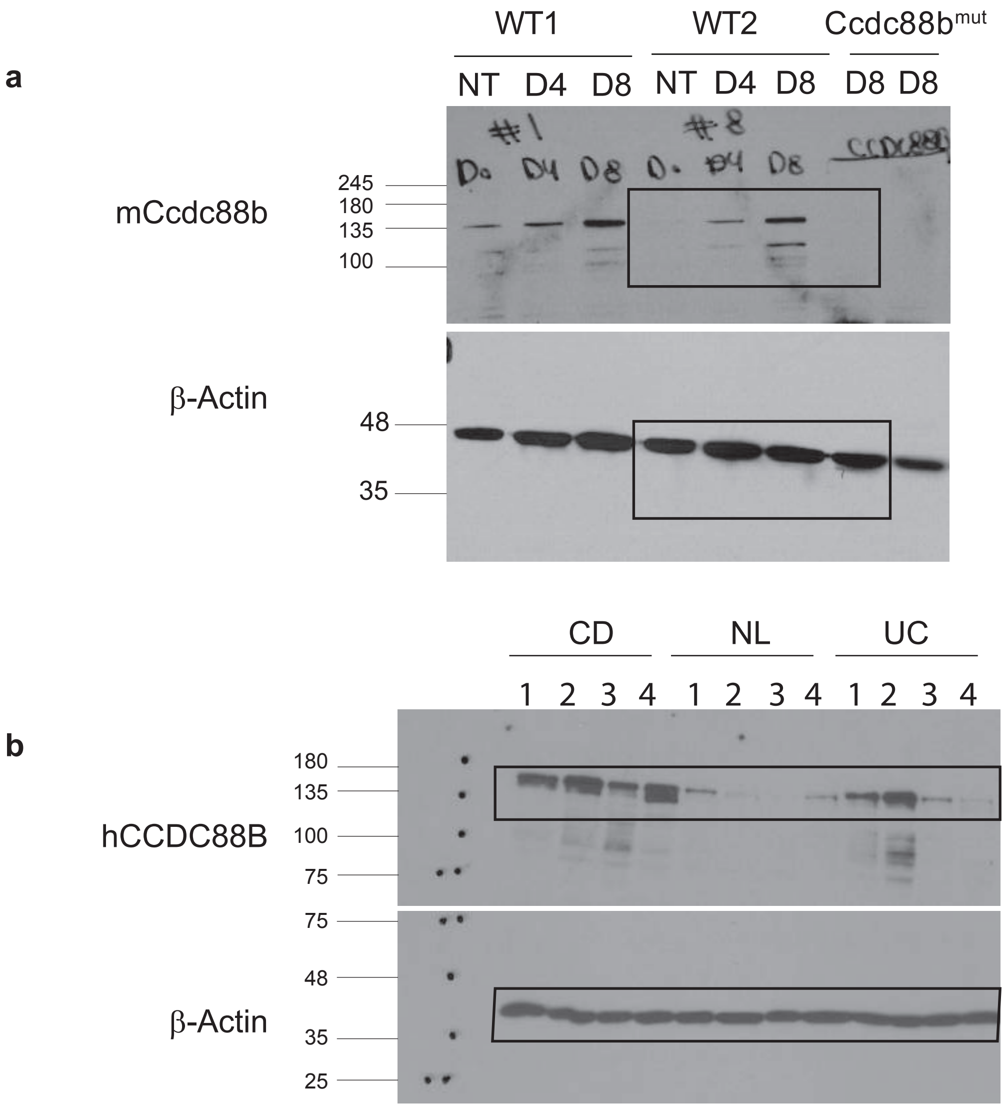

Supplementary Figure 7: Uncropped blot images of western blots analyses in Figure 1 and Figure 6.

(a)) Figure 1b, immunoblot analysis of 2 replicate depicting Ccdc88b protein expression in mice colon from WT 1 (control) and WT2 (control) at day 4 and day 8 post-treatment, and of 2 *Ccdc88b* mutant mice (at day 8), with corresponding immunoblots for actin. (b), Figure 6b. In each panel, rectangles represent cropped images used in the Figure 6b and that include tissue samples from normal controls (NL), Crohn's (CD) and ulcerative colitis (UC) patients, with corresponding actin controls.

Supplementary Table 1: Assessment of Intestinal Inflammation

Scoring	Inflammatory cell infiltration
0	Occasional resident inflammatory cells in lamina propria (predominantly lymphocytes, plasma cells)
1	Minimal increase in inflammatory cells
2	Mild increase in inflammatory cells
3	Moderate increase in inflammatory cells
4	Marked increase in inflammatory cells
**Scoring**	**Inflammatory cell type**
L	Significant presence of lymphocytes and plasma cells
M	Significant presence of macrophages
G	Significant presence of multinucleate giant cells
N	Significant presence of neutrophils
E	Significant presence of eosinophils
**Scoring**	**Inflammatory cell depth**
0	No significant inflammatory infiltration
1	Inflammatory infiltration predominantly in the lamina propria
2	Inflammatory infiltration extending significantly into the submucosa
3	Inflammatory infiltration extending significantly into the muscularis
4	Inflammatory infiltration extending significantly to the serosa/mesentery
**Scoring**	**Mucosal thickening**
0	Normal mucosal thickness
1	Small areas of mucosal thickening
2	Extensive areas of mild thickening
3	Extensive areas of marked thickening
4	Diffuse marked thickening
**Scoring**	**Surface epithelial degeneration**
0	Normal surface epithelium
1	Rare, small areas of surface epithelial flattening, rounding or sloughing off (<25% of surface)
2	Occasional small or few mildly extensive areas of surface epithelial flattening, rounding or sloughing off (25-50% of surface)
3	Frequent small or occasional moderate areas of surface epithelial flattening, rounding or sloughing off (50-75% of surface)
4	Extensive areas of surface epithelial flattening, rounding or sloughing off (>75% of surface)
**Scoring**	**Gland epithelial apoptosis**
0	Absent or very rare apoptotic cells in the gland epithelium
1	Slightly increased number of apoptotic cells in gland epithelium
2	Mildly increased number of apoptotic cells in gland epithelium
3	Moderately increased number of apoptotic cells in gland epithelium
4	Markedly increased number of apoptotic cells in gland epithelium
**Scoring**	**Gland epithelial degeneration/abscesses**
0	No glands showing degeneration or abscesses
1	Rare glands showing degeneration or abscesses
2	Occasional glands showing degeneration or abscesses
3	Frequent glands showing degeneration or abscesses
4	Most glands showing degeneration or abscesses
**Scoring**	**Gland goblet/enterocyte ratio decrease**
0	Normal ratio of goblet to enterocyte throughout all glands
1	Rare areas of glands showing increased proportion of enterocytes to goblet cells
2	Occasional areas of glands showing increased proportion of enterocytes to goblet cells
3	Frequent areas of glands showing increased proportion of enterocytes to goblet cells
4	Most of section with glands showing increased proportion of enterocytes to goblet cells
**Scoring**	**Submucosal edema**
0	No significant submucosal edema
1	Rare areas of mild submucosal edema
2	Occasional areas of mild, or rare areas of moderate submucosal edema
3	Frequent areas of mild to moderate submucosal edema
4	Frequent areas of marked submucosal edema
**Scoring**	**Gland loss**
0	Normal density of glands
1	Rare foci of gland loss, over small areas (<25% of surface)
2	Occasional small foci of gland loss, or rare but wider foci (25-50 % of surface)
3	Frequent small foci of gland loss, or occasional wide foci (50-75 % of surface)
4	Extensive gland loss, over most of the mucosa (>75 % of surface)
**Total pathology score: sum of all scores**	
**Injury score: inflammatory cell infiltration, surface epithelial degeneration and gland loss**	
